# Recurrent Laryngeal Nerve Schwannoma: A Case Report

**DOI:** 10.7759/cureus.92049

**Published:** 2025-09-11

**Authors:** Komalpreet Kaur, John R LaFrentz

**Affiliations:** 1 Medicine, Edward Via College of Osteopathic Medicine, Auburn, USA; 2 Otolaryngology - Head and Neck Surgery, Huntsville Ear, Nose &amp; Throat Physicians, P.C., Huntsville, USA

**Keywords:** neck tumor, neuroma, recurrent laryngeal nerve, schwannoma, thyroid tumor

## Abstract

Generally, less than half of neurogenic tumors are found in the head and neck region. Within that distinction, the majority of them originate from a branch of the superior laryngeal nerve in the neck. Schwannomas are benign tumors arising from Schwann cells, which form the myelin sheath of peripheral nerves. In the medical literature, evidence of recurrent laryngeal nerve schwannomas is sparse.

This report describes the case of a 77-year-old woman with a history of multinodular goiter and primary hyperparathyroidism. During parathyroid exploration, an isolated schwannoma was incidentally found arising from the left recurrent laryngeal nerve. The lesion was confirmed by intraoperative nerve stimulation. To avoid vocal cord paralysis, the tumor was not resected. The patient was managed conservatively and remained asymptomatic over six years, with stable tumor size on imaging.

This case highlights the importance of identifying rare neurogenic tumors during neck surgery and weighing surgical risks against functional outcomes.

## Introduction

Neurogenic tumors (neuromas) are benign tumors that arise from nerve cells found in the peripheral nervous system. They are further classified into schwannomas and neurofibromas. Schwannomas account for approximately 25% to 45% of neurogenic tumors occurring in the head and neck region [1]. These tumors commonly originate from branches of the superior laryngeal nerve, a branch of the vagus nerve. Around 10% of schwannomas arise from the vagal or sympathetic nervous systems [2]. Schwannomas of the recurrent laryngeal nerve are rare. Histologically, schwannomas arise from Schwann cells only, whereas neurofibromas arise from Schwann cells and fibroblasts [3,4].

Though benign, schwannomas can have functional consequences depending on their location. Treatment options include surgical excision or observation. Excision carries a risk of vocal fold paralysis, while observation is appropriate in asymptomatic cases. Life expectancy is not affected, but the choice of management can impact voice and airway function [5].

This case highlights a schwannoma originating from the recurrent laryngeal nerve. It should be recognized for having implications for physicians and patients regarding treatment and prognosis since there are only a handful of such cases found in the literature.

## Case presentation

**Figure 1:**
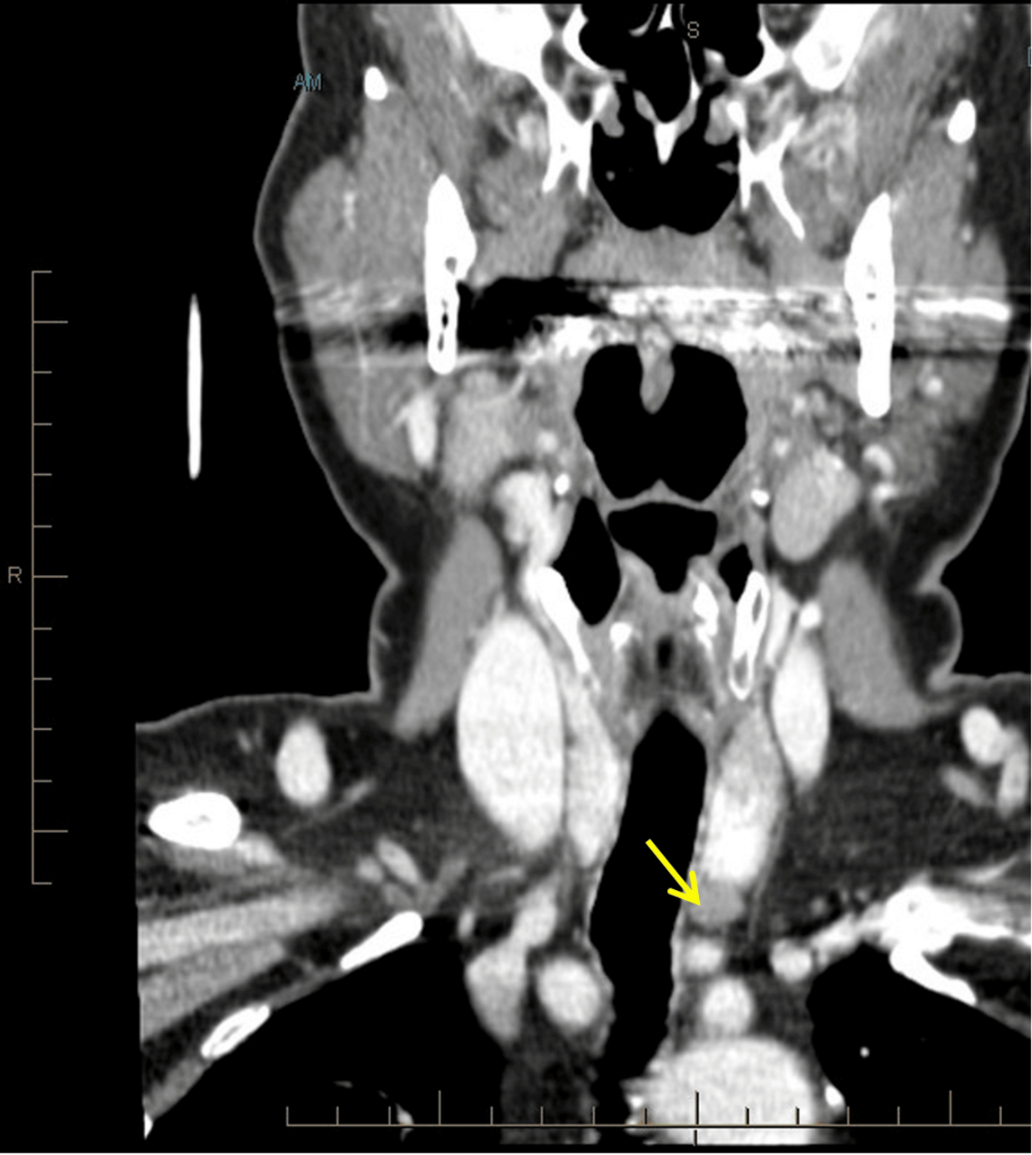
Neck CT scan with contrast. The yellow arrow indicates the schwannoma, measuring 1.1 cm.

A 77-year-old woman with a past medical history of non-toxic multinodular goiter presented with a painless neck mass and globus sensation. She was noted to have multinodular goiter and hypercalcemia, with a calcium elevation greatest at 12.9 mg/dL and an elevated parathyroid hormone of 156 pg/mL. Thyroid ultrasound detected a 2-cm solid nodule within the right aspect of the isthmus and a smaller solid nodule in the superior pole of the left lobe of the thyroid, both Bethesda category II on fine needle aspiration. A contrast-enhanced computed tomography (CT) scan of the neck with parathyroid protocol demonstrated a 1.1-cm nodule adjacent to the posterior-inferior margin of the thyroid lobe thought to be a parathyroid adenoma (Figure 1). On imaging, the thyroid gland was visualized bilaterally adjacent to the trachea. It demonstrated a heterogeneous or coarse attenuation pattern on the CT scan. The trachea appeared as a vertically oriented, air-filled tubular structure with low attenuation (black) on coronal sections.

**Figure 2:**
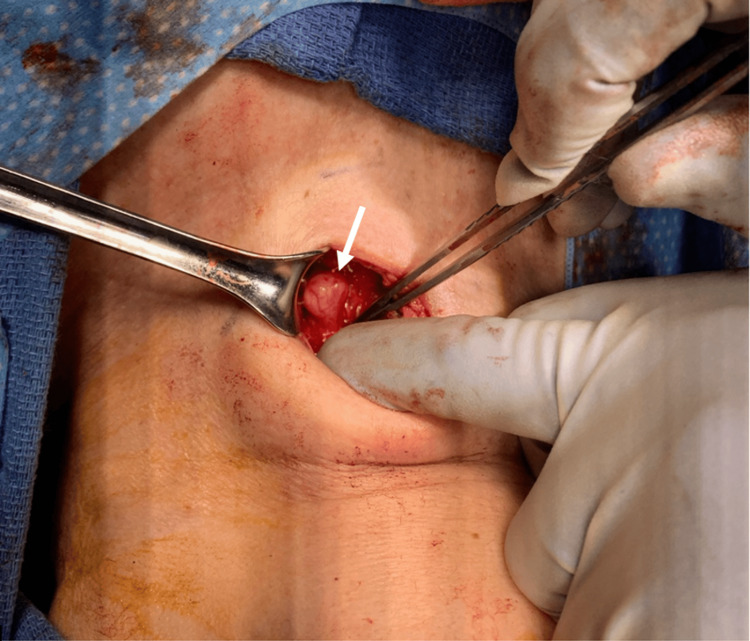
Intraoperative image of the recurrent laryngeal nerve schwannoma. The white arrow indicates the schwannoma.

The patient was scheduled for parathyroid surgery. At the suspected location of the left inferior parathyroid adenoma, a small neuroma was identified. The neuroma was stimulated with the nerve integrity monitor and was thought to be a left recurrent laryngeal nerve schwannoma (Figure 2). The decision was made to leave the neuroma undisturbed as resecting the neuroma would have led to permanent left true vocal fold paralysis. A four-gland exploration was performed, resulting in the resolution of primary hyperparathyroidism.

## Discussion

Neuromas are tumors of neurogenic origin and can be subclassified as schwannomas and neurofibromas [3]. A key differentiating factor between the two is that schwannomas only arise from Schwann cells and are well-encapsulated and grow extrinsically to the nerve fascicles, whereas neurofibromas can arise from fibrocytes and/or Schwann cells and are not encapsulated and grow intertwiningly with the nerve fascicles [3,4]. Schwannomas are much more common [1]. In this case, the neuroma was a well-circumscribed, solitary lesion growing intrinsic to the left recurrent laryngeal nerve. Additionally, neurofibromas are usually not solitary lesions but rather multiple lesions along the whole length of the nerve fibers. Neurofibromas are associated with neurofibromatosis, which our patient did not have [1,6]. Although the tumor was not excised, which represents a limitation of this report, the lesion's intraoperative characteristics are most consistent with a presumed schwannoma.

It is uncommon for these neck masses to be symptomatic, and patients can present with dysphonia and dyspnea in the fourth or fifth decade of life. Additional symptoms include globus sensation, odynophagia, dysphagia, and complete airway obstruction [1,5,6]. Although the origin is sporadic, there is a sex predilection for women in schwannomas [6]. Our patient complained of globus sensation but no hoarseness or change to her voice quality. The globus sensation could also be explained by the isthmus nodule.

Based on the size and the benign, slowing-growing nature of the tumor, and the fact that the patient did not complain of hoarseness or dyspnea, we decided not to excise the tumor to prevent vocal cord paralysis.

The gold standard for diagnosis is histology, as imaging cannot reliably distinguish between schwannomas and neurofibromas [5]. Schwannomas are well-encapsulated spindle cell tumors with alternating hypercellular Antoni A and hypocellular Antoni B areas. They show strong, uniform immunoreactivity for S-100 and SOX-10. In contrast, neurofibromas are unencapsulated, composed of loosely arranged spindle cells in a fibro-myxoid matrix, and demonstrate patchy S-100 positivity with variable CD34 and EMA expression [7].

**Figure 3:**
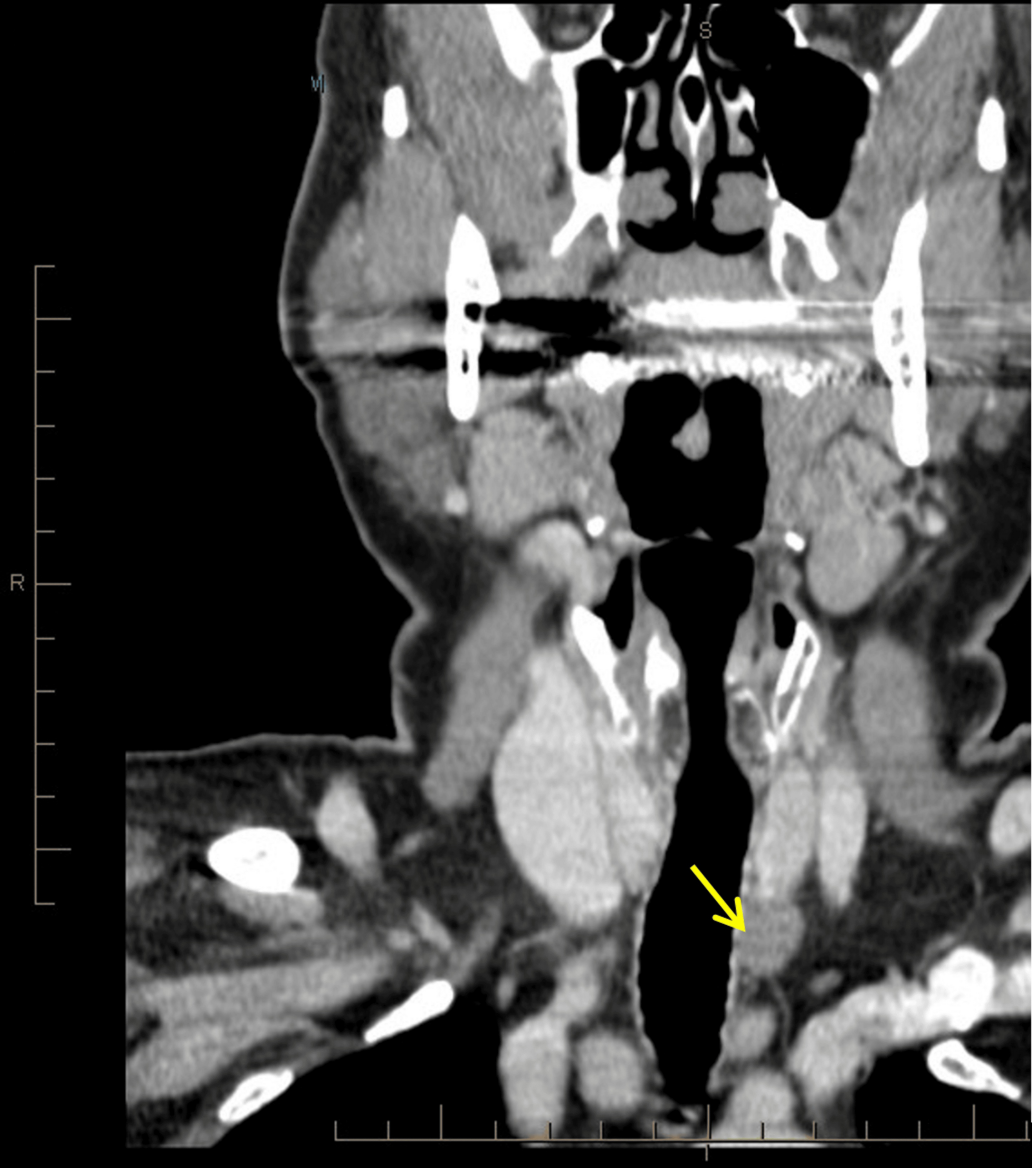
Follow-up neck CT scan with contrast. The yellow arrow indicates the schwannoma three years after the original scan.

The patient’s management course is watchful waiting with annual imaging to ensure stability of the schwannoma (Figure 3). After mild initial growth in the first year, the size remains relatively stable at 1.5 cm on most recent ultrasound. The patient continues to be asymptomatic. Similarly, Kim et al. pursued this course of action without complications [8].

## Conclusions

Isolated neuromas originating from the recurrent laryngeal nerve are a rare occurrence in the head and neck region, while laryngeal tumors from the superior laryngeal nerve are more common. This case of a 77-year-old woman with a recurrent laryngeal nerve schwannoma highlights the challenges in diagnosing such lesions, as they often mimic other common conditions, such as parathyroid adenomas or thyroid nodules. While surgical intervention is an acceptable treatment for neurogenic tumors in the neck, the decision to leave the neuroma undisturbed in this case was made to avoid the risk of vocal fold paralysis. In asymptomatic and stable lesions, particularly involving the recurrent laryngeal nerve, watchful waiting with regular monitoring may be appropriate. This approach proved to be a viable management option for this patient, who remained asymptomatic over six years. This case adds to the growing body of literature to ensure the best outcomes for patients with rare neurogenic tumors in the neck region.
